# Class-I and Class-II Restorations with the Application of a Flowable Composite as an Intermediate Layer—A Narrative Review of Clinical Trials

**DOI:** 10.3390/jfb16030111

**Published:** 2025-03-20

**Authors:** Anh Duc Nguyen, Kerstin Bitter, Christian Ralf Gernhardt

**Affiliations:** University Outpatient Clinic for Conservative Dentistry and Periodontology, Department of Dental Medicine, Medical Faculty, Martin-Luther-University Halle-Wittenberg, Magdeburger Strasse 16, 06112 Halle, Germany

**Keywords:** adhesive dentistry, cavity lining, composite restoration, posterior restorations, flowable composite, clinical trials, clinical success, restorative dentistry

## Abstract

The objective of this review is to investigate the effect of an additional layer of flowable composite for cavity lining on the clinical outcome of direct posterior composite restorations. The PICO question (patient, intervention, comparison, and outcome) was stated as follows: Does the additional application of a flowable composite as a cavity liner improve the clinical outcome of Class-I and Class-II restorations? The electronic databases MEDLINE, Web of Science, LILAS, and BBO were assessed for identifying relevant clinical studies. After removal of duplicate records, 309 records could be identified and, after a screening of the title and abstract, 20 articles were selected for full-text analysis. Finally, six studies met the eligibility criteria and were included in this review for further investigation. Four of the included studies have a follow-up period of two years, while the other two studies had an observation period of three and seven years, respectively. No significant differences in annual failure rates were observed between restorations with and without a flowable composite liner. Consequently, the additional usage of flowable composites as a cavity liner seems to have no effect on the clinical longevity of direct composite restorations in Class-I and Class-II cavities. Therefore, the application of a flowable composite is a possible option in everyday dental clinical practice.

## 1. Introduction

The practice of operative dentistry remains a fundamental aspect of modern dentistry and constitutes a substantial component of the oral healthcare. A considerable part of operative dentistry encompasses the prevention and diagnosis of dental caries and the restoration of teeth requiring treatment due to the loss of dental hard tissue. The application of composite materials in the discipline of adhesive dentistry has thus become a well-established practice for direct restoration procedures in anterior and posterior teeth [1,2]. The aforementioned restorations are in accordance with the expectations of both the patient and the practitioner with regard to an esthetically pleasing and minimally invasive therapeutic concept [3]. Leakage, marginal adaptation and subsequent secondary caries remain considerable concerns with composite restorations, particularly on the proximal region of posterior teeth restorations. Insufficient marginal sealing has been identified as a potential contributing factor in postoperative sensitivity, restoration failures, and secondary caries [4]. Therefore, some authors recommend using flowable composites as a cavity lining material to increase internal adaption. Moreover, the primary objective in the application of flowable composites as cavity liners is the prevention of occlusal leakage, particularly in the approximal region of deep carious lesions [5]. A subset of in vitro studies has demonstrated the efficacy of a flowable liner under composite restorations in mitigating marginal adaption and reducing microleakage [6,7,8]. This finding stands in contrast to the results observed in other studies [9,10,11,12]. Moreover, the handling of flowable composites is reportedly advantageous due to their reduced viscosity during application [13,14].

**Figure 1:**
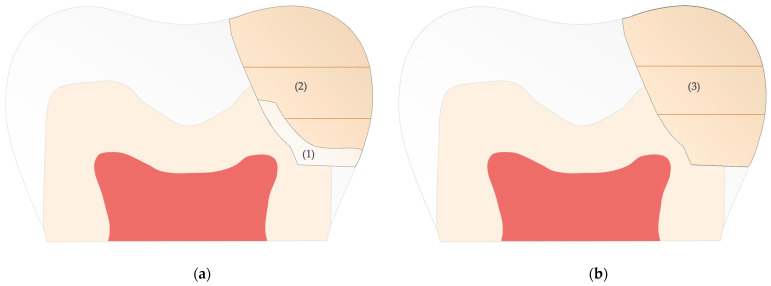
(**a**) A schematic illustration of a tooth restored by receiving a cavity lining with a flowable composite (1) and the subsequent application of the composite material using an increment technique (2). Structures: enamel (white), dentine (yellow), pulp (red). (**b**) A schematic illustration of a tooth restored without a cavity lining and only with the application of the composite material using an increment technique (3). Structures: enamel (white), dentine (yellow), pulp (red).

Accordingly, some authors have advised the additional use of a flowable composite for the lining of cavities (Figure 1), with a view to minimizing polymerization stress and enhancing the adaptation of the adhesive filling materials [15,16]. The defining characteristic of flowable composites is their reduced proportion of filler particles, which results in a reduction in their viscosity [13]. Consequently, flowable composites exhibit an elastic modulus that is 20–30% less than that observed in conventional composite materials [17,18]. The diminished elasticity modulus endows low-viscosity flowable composites with the capacity to absorb stress induced by polymerization shrinkage and mechanical loading on the teeth during their functional use [19]. Furthermore, they are distinguished by their enhanced handling during the clinical application of the filling [20].

The results of a survey of dental practitioners in Germany indicate that 80.1% of the 1449 participating dentists utilize a flowable composite for cavity lining [21]. The current state of research on the impact of flowable composite materials on the long-term outcomes of composite restorations is fragmented and lacks a comprehensive consensus [6,11,15,22]. Although the scientific evaluation of these materials is still limited, there has been a notable rise in the use of flowable composites as a cavity liner in the treatment of damaged teeth with carious and non-carious lesions [23,24,25,26,27]. Nonetheless, there is currently a controversy in the literature regarding the additional application of a flowable composite liner under composite restorations. There is currently no consensus as to whether a cavity liner is advantageous for the adhesive restoration of Class-I and Class-II cavities [6,11,28,29,30]. For this reason, the aim of this review is to provide an overview of the longevity and outcome of composite restorations in order to compare this debate in the clinical setting. A number of studies have been performed on bulk-fill composites, which are distinguished by their higher layer thickness of approximately 4 mm per increment during application in comparison to other flowable composite materials [5,15,31,32,33,34]. Bulk-fill restorative materials have been widely integrated into clinical practice due to their ability to enhance the efficiency of dental treatments by reducing procedural time, while also demonstrating favorable esthetic properties, making them a valuable advancement in contemporary restorative dentistry [35]. The authors of the present review chose to exclude this group of materials, as they exhibit different workability during placement of the restorations due to their chemical composition, which could make a comparison of the studies with conventional methacrylate-based composites difficult.

The aim of the present review is to ascertain whether the additional application of flowable composites as a cavity liner affects the clinical outcome of direct restorations in the posterior region of Class-I and Class-II cavities. For this purpose, only clinical trials are considered for the selection of studies. The null hypothesis stated that the additional cavity lining of Class-I and Class-II has no impact on the longevity and marginal integrity of composite restorations compared to restorations without an intermediary layer of flowable composites.

## 2. Materials and Methods

### 2.1. PICO Question

The following research question was developed to guide the search strategy: Does the additional application of a high-viscosity flowable composite as a cavity liner improve the clinical outcome of Class-I and Class-II restorations?

In accordance with the PICO framework, the population comprised patients with direct composite restorations of Class-I or Class-II cavities in permanent teeth. The intervention involved the application of a flowable composite as an intermediate layer in Class I or Class II cavities, while the comparison was made with composite restorations that did not include an intermediate layer. The outcomes were evaluated in terms of restoration failures observed in the clinical studies.

### 2.2. Search Strategy

The electronic databases MEDLINE, Web of Science, LILAS, and BBO were assessed for identifying relevant clinical studies. A comprehensive search of all databases was performed on 26th October 2024. No restrictions were imposed with respect to the publication medium, the publication date, or the language. The search strategy was developed on the basis of the initially formulated PICO question. Therefore, we used the following search strategy for Medline (via PubMed):

(((((((((((((occlu*) OR occlu* proximal) OR class I cavitie*) OR class II cavitie*) OR class I) OR class II) OR approximal lesion*) OR proximal lesion*) AND composite [MeSH Terms]) OR composite*) OR resin* composite) OR conventional composite*)) AND ((((((((((occlu* proximal) OR class I cavitie*) OR class II cavitie*) OR approximal lesion*) OR proximal lesion*) AND flowable hybrid composite[MeSH Terms]) OR flowable hybrid composite) OR flowable composite) OR flow line) OR flowable resin*) AND (((((cavity lining) OR cavity liner) OR intermediate layer) OR intermediary layer) OR liner).

This search strategy was then adapted for the Web of Science database and the following search strategy was applied:

((TS = (flowable composite OR flowable hybrid composite OR flowable OR flow line)) AND TS = (occlu* OR occlu* proximal OR class I cavitie* OR class II cavitie* OR class I OR class II OR approximal lesion* OR proximal lesion*)) AND TS = (cavity lining OR cavity lining OR intermediate layer OR intermediary layer OR liner).

Furthermore, the two electronic databases LILACS and BBO were assessed for the screening of relevant articles according to our inclusion criteria. Therefore, the following terms were used for search strategy: ((cavity lining) OR (cavity liner) OR (intermediate liner) OR (intermediate lining)) AND (flow* composite).

### 2.3. Study Selection

The records from MEDLINE and Web of Science databases were collected in a reference management software (EndNote™ Version 20.6, Clarivate™ Analytics, Philadelphia, PA, USA), and any duplicates were subsequently removed. Two authors of the review (A.D.N., C.R.G.) then evaluated the titles and abstracts of the studies in accordance with the established eligibility criteria. Studies not matching the inclusion criteria or which clearly had no relevance for the review were then excluded. Subsequently, the authors checked the full text of the potentially relevant studies against the eligibility criteria, determining their inclusion or exclusion in the review. Furthermore, the references were screened during the full-text analysis to guarantee that potentially relevant literature was not missed. In the case that follow-up studies have been published at varying time points, the most recent investigation with the longest period is considered. Finally, the studies selected by both authors were included in the review.

### 2.4. Inclusion/Exclusion Criteria

The following table summarizes all inclusion and exclusion criteria of published clinical trials for selection progress (Table 1).

## 3. Results

**Figure 2:**
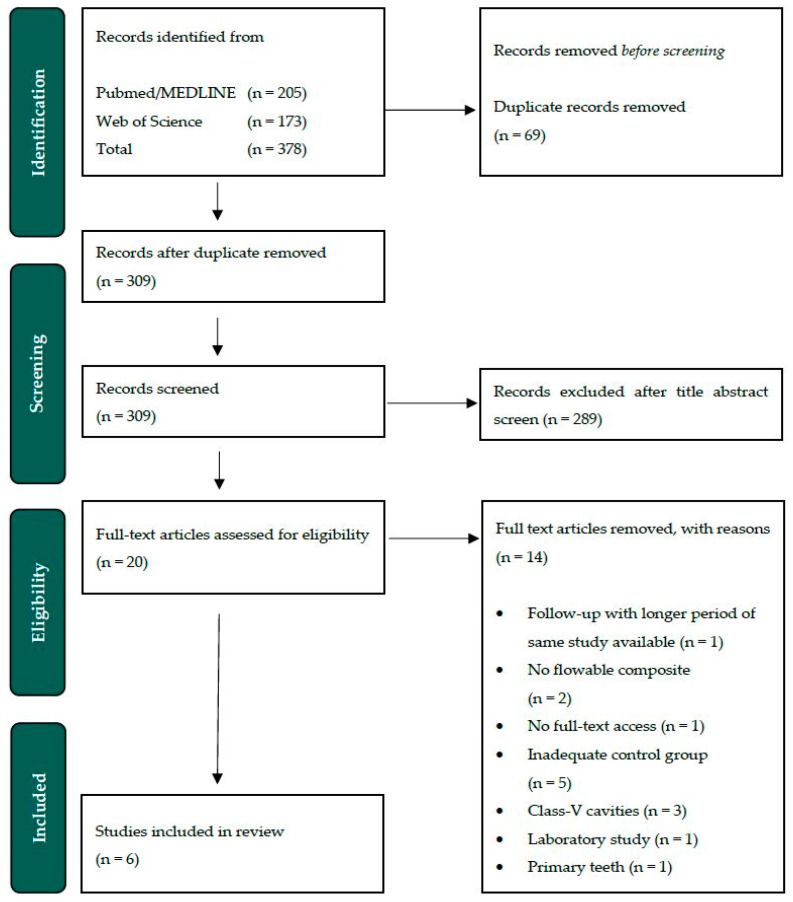
A schematic illustration of the used search strategy and final study-selection process according PRISMA guidelines for reviews [42].

The search strategies identified 309 records after the removal of duplicates. After an examination of the title and abstract, 289 records were excluded, and the remaining 20 articles were checked for eligibility by analyzing the full text. A further 14 studies were excluded for various reasons, which are summarized in Figure 2.

A hand search in LILACS and BBO databases and a screening of the references during full-text analysis could not detect further relevant studies for inclusion according to our eligibility criteria. Finally, 6 studies were included for the present review (Figure 2): 5 randomized clinical studies and 1 clinical trial with no random allocation employed.

Of these included studies, 4 had a follow-up period of 2 years [36,37,38,39], 1 had a follow-up period of 3 years [40], and the last study had a follow-up period of 7 years [41]. The main characteristics concerning study type and setting, follow-up periods, cumulative survival rate, and the annual fracture rate (AFR) were summarized in Table 2. Moreover, the tested composite materials and adhesive systems as well as their conditioning mode are listed in Table 2.

**Table 2:** Characteristics and outcomes of all included clinical trials of this review.

Study	Study Design/Setting	Follow-Up [Years]	No. Patients (Restorations) at Baseline	Recall Rate	Cumulative Survival Rate	AFR	Rubber Dam Isolation	Restorative Materials	Adhesive System [Modulus]	Assessment Criteria
Boeckler et al., 2012 [36]	Split-mouth/university	2	50 (100)	88%	F: 100% C: 97.7%	F: 0% C: 1.2%	yes	FL: Tetric Flow ^1^ CR: Tetric Ceram ^1^	AdheSE One ^1^ [SE]	Modified USPHS/Ryge
Efes (1) et al., 2006 [37]	Split-mouth/university	2	27 (54)	96%	F: 100% C: 100%	F: 0% C: 0%	no	FL: Filtek Flow ^2^ CR: Filtek Supreme ^2^	Single Bond ^2^ [ER]	Modified USPHS/Ryge
Efes (2) et al., 2006 [37]	Split-mouth/university	2	27 (54)	89%	F: 100% C: 96%	F: 0% C: 2%	no	FL: Admira Flow ^3^ CR: Admira ^3^	Admira Bond ^3^ [ER]	Modified USPHS/Ryge
Ernst et al., 2003 [38]	Split-mouth/university	2	50 (116)	95%	F: 92.8% C: 94.6%	F: 3.6% C: 2.7%	70%	FL: Revolution ^4^ CR: Prodigy ^4^	Optibond Solo Plus ^4^ [ER]	USPHS/Ryge
Nguyen et al., 2024 [40]	Split-mouth/university	3	50 (100)	92%	F: 91.3% C: 100%	F: 2.9% C: 0%	yes	FL: GrandioSO Heavy Flow ^3^ CR: GrandioSO ^3^	Futurabond DC ^3^ [SE]	Modified USPHS/Ryge
Stefanski et al., 2012 [39]	Split-mouth/university	2	48 (96)	96%	F: 97.8% C: 97.8%	F: 1.1% C: 1.1%	no	FL: Filtek Flow Supreme XT ^2^ CR: Filtek Supreme XT ^2^	Adper Scotchbond 1 XT ^2^ [ER]	Modified USPHS/Ryge
Van Dijken et al., 2011 [41]	Split-mouth/university	7	48 (118)	96%	F: 86% C: 84.2%	F: 2% C: 2.3%	no	FL: Tetric Flow ^1^ CR: Tetric Ceram ^1^	Excite ^1^ [ER]	Modified USPHS/Ryge

Abbreviations: No. = number; F = restoration group with cavity lining; C = restoration group without cavity lining; FL = flowable composite liner; CR = packable composite resin; AFR = annual failure rate; SE = self-etch mode; ER = etch-and-rinse mode. ^1^ Ivoclar Vivadent GmbH, Ellwangen, Liechtenstein. ^2^ 3M ESPE, St. Paul, MN, USA. ^3^ VOCO GmbH, Cuxhaven, Germany. ^4^ Kerr Corporation, Orange, CA, USA.

## 4. Discussion

This review aims to evaluate the clinical outcome of direct composite restorations placed with an intermediary layer of flowable composites as cavity lining material in Class-I and Class-II cavities. Although the use of flowable composites in clinical practice is very common [21], the number of available clinical trials suitable for the present review is considerably limited. Only six studies met the inclusion criteria and were included into the review.

### 4.1. Methodology

All of the included studies used the USPHS/Ryge-criteria [43] for evaluation of the clinical outcome of the placed direct posterior restorations. The following parameters were applied by all authors for the assessment at baseline and the follow-up investigations: secondary caries, surface texture, marginal adaption, marginal discoloration, and color match (Table 3). The parameters tooth vitality, postoperative sensitivity, proximal contact, and anatomic form were not covered by all authors. The observation period in four of the studies [36,37,38,39] was limited to two years, which is a relatively brief period for identifying differences between the test and control groups. A further limitation is the paucity of six studies meeting the inclusion criteria, which makes it difficult to draw conclusions for clinical application due to the short observation period and the small number of included studies.

**Table 3:** A summary of the failure reason of parameters that were evaluated in all studies [%]; F = restoration group with cavity lining; C = restoration group without cavity lining; n.r. = not reported.

Parameter		Anatomical Form	Secondary Caries	Tooth Vitality	Filling Integrity	Surface Roughness	Marginal Adaption	Marginal Discoloration	Color Match
Study/ Group									
Boeckler et al. [36]	F	n.r.	0	0	0	0	0	0	0
	C	n.r.	0	0	0	0	2.3	0	0
Efes (1) et al. [37]	F	n.r.	0	n.r.	0	0	0	0	0
	C	n.r.	0	n.r.	0	0	0	0	0
Efes (2) et al. [37]	F	n.r.	0	n.r.	0	0	0	0	0
	C	n.r.	0	n.r.	4.2	0	0	0	0
Ernst et al. [38]	F	5.5	1.8	1.9	n.r.	5.5	5.5	0	0
	C	3.6	0	0	n.r.	3.6	3.6	0	0
Nguyen et al. [40]	F	n.r.	0	6.5	4.3	0	0	0	0
	C	n.r.	0	0	0	0	0	0	0
Stefanski et al. [39]	F	2.2	0	n.r.	n.r.	0	0	0	0
	C	2.2	0	n.r.	n.r.	0	2.2	0	0
Van Dijken et al. [41]	F	8.8	3.5	n.r.	n.r.	0	8.8	0	0
	C	12.3	5.3	n.r.	n.r.	0	12.5	0	0

One study investigated only Class-I cavities [37], three studies only Class-II cavities [38,39,41] and two studies included both cavity Classes I and II [36,40]. Also, the first mentioned study [37] only included maxillary molars, whereas the remaining five studies [36,38,39,40,41] included premolars and molars located in both jaws.

The published clinical trial of Ernst et al. [38] did not perform a random allocation of the selected teeth to the different experimental groups, which could result in decreased internal validity [44]. Furthermore, a high number of dentists (n = 10) were involved in the restoration process. Although they had undergone a calibration process on phantom models beforehand, it is challenging to compare the restoration quality in a clinical setting. This may be a contributing factor in the clinical outcome when considering the higher AFR in both tested groups compared to the other studies, which remains within clinically acceptable and comparable ranges. The remaining five studies had a maximum of only two calibrated operators and examiners, with only one study failing to report blinding [39]. The examiners for the follow-up evaluations of the other four studies were not involved in the clinical restoration process [36,37,40,41], decreasing the risk of bias.

### 4.2. Used Composite Materials

In our review, we solely included studies using conventional methacrylate-based composite materials and excluded studies using glass ionomer cement, provisional restorative materials, and bulk-fill composite resins (Table 1). The study of Efes et al. [37] investigated both material groups, a nanofilled methacrylate composite resin and an ormocer-based composite material. We included this study due to the study design, methodology, and clinical procedure of the restorations, as well as the comparable parameters evaluated. Ormocer-based composites were also considered, although they have other physical and chemical properties compared to methacrylate-based composite resins [45,46].

In one study [40], a so-called high-viscous or heavy flowable composite was used for cavity lining. This group of materials can be used as an alternative to regular viscous flowable composites, as it demonstrated comparable outcomes in several in vitro publications [17,47]. It is characterized by its higher filler content and modulus of elasticity compared to regular viscosity flowable composites [48]. While numerous clinical studies have examined the combination of conventional flowable composites with packable composites, the number of clinical studies investigating high-viscosity flowable composites remains limited and is controversial. In accordance with the results of the clinical trial of Ñaupari-Villasante et al. [49], the high-viscosity flowable composites showed significantly higher failure rates than the low-viscosity flowable composites and ormocer-based flowable composites when used for cavity lining after an observation period of 48 months. In contrast, Torres et al. [50] demonstrated that neither low-viscous nor high-viscous flowable composites exhibited superior clinical outcomes, which align with the results of Nguyen et al. [40]. A comparison of the AFR with studies using regular viscosity flowables [36,37,38,39,41] indicates that there is no discernible impact of viscosity on the longevity of restorations, as the AFR seems to have clinical acceptable values.

In our study-selection process, we implemented no restrictions concerning the used adhesive system of the clinical trials. In the field of modern adhesive dentistry, bonding systems are typically classified into two categories, depending on the mechanism of dissolution employed to remove the smear layer [51]. The etch-and-rinse adhesives require a 35–40% phosphoric acid gel for preconditioning enamel and dentin before applying the bonding agent [52]. The additional phosphoric acid application is not mandatory when using self-etch adhesives due to their ability of conditioning enamel and dentin using acidic monomers [53]. Nonetheless, studies have demonstrated superior bond strength values for self-etch adhesive systems when these were previously conditioned with phosphoric acid in the enamel area [30,54].

Recent studies have demonstrated increased marginal integrity when the enamel of the cavity was conditioned with phosphoric acid prior using the adhesive material, which is associated with a reduction in marginal discoloration [30,55,56,57]. The study of Boeckler et al. [36] using a self-etch adhesive showed less occurrence of marginal discoloration at the 2-year follow-up, whereas a notable increase in Code Bravo rating was observed by Nguyen et al. [40] after 3 years compared to the evaluation after 2 years. In comparison to Stefanski et al. [39] and Ernst et al. [38], where etch-and-rinse adhesives were used as bonding agent comparable results were evaluated after 2 years. However, there are no existing studies that have investigated the effect of an intermediate liner in composite restorations on marginal discoloration and the used adhesive system. Regarding the results of all included studies concerning marginal discoloration, no restoration was rated as a failure (Table 3).

### 4.3. Clinical Procedure

Three of the included studies perform an isolation of the operative field using a suction device and cotton rolls during the placement of filling materials [37,39,41]. In contrast, rubber dam isolation was used for two other studies [36,40], and one study performed absolute isolation in 70% of the cases [38]. A recently published clinical in situ study of Falacho et al. [58] showed superior outcomes for adhesive restorations placed under rubber dam isolation, particularly with regard to the humidity control of enamel and adhesive bond strength. However, a 2014 review by Cajazeira et al. [59] indicated that isolation with a rubber dam has no effect on the longevity of restorations. Nevertheless, it seems that there is no discernible impact on the clinical outcome of the studies with respect to the cumulative success rate and AFR (Table 2).

Three studies [38,39,40] performed indirect capping [60,61] in deep carious lesions, with one study using a glass-ionomer-cement base [38] and the other two using calcium hydroxide [39,40]. The inclusion of teeth with deep carious lesions indicating indirect pulp capping is more closely aligned with the clinical setting of dental practice. Accordingly, one dropout was recorded in the study of Ernst et al. [38] and three failures in the study of Nguyen et al. [40] due to loss of vitality. Stefanski et al. [39] performed indirect pulp capping, yet tooth vitality was not subjected to any form of assessment. Two additional studies likewise did not conduct tooth vitality tests during the follow-up evaluations, analogous to Stefanski et al. [39], which eliminates a criterion for a dropout. Consequently, the failure rate may be higher than indicated (Table 2). Boeckler et al. [36] did not observe any loss of tooth vitality in the teeth examined in their clinical trial.

Four studies provided data about the layer thickness of their used liner material: one study [40] applied a 0.5 mm layer, another study [37] 1 mm, and two studies [39,41] 1–1.5 mm. The results of two in vitro studies indicated a correlation between increasing liner thickness and a decline in marginal integrity [62,63]. In contrast, a meta-analysis yielded no discernible effect of whether the flowable layer thickness was below or above 2 mm [64]. Ernst et al. [38] recorded more dropouts in both tested groups due to unacceptable scores of the marginal adaption (Table 3) compared to the other studies with a 2 year-follow-up. In the absence of data regarding layer thickness, it is not possible to draw any conclusions regarding the correlation between thickness and marginal quality. Van Dijken et al. [41] also reported a high percentage of unacceptable scores in marginal adaption in both groups (with flowable liner: 8.8%, without flowable liner: 12.5%) applying a 1–1.5 mm thick layer of the lining material. In comparison, Stefanski et al. [39] observed no instances of failure due to a lack of marginal adaptation in the group that used a flowable liner, which had the same thickness for cavity lining. However, one tooth in the other group was rated as a failure due to lack of marginal integrity, which aligns with the results of the meta-analysis [64], that the layer thickness does not correlate with bond strength and as a result with the marginal adaption of the restoration.

This review has several strengths, such as the inclusion of clinical trials with an observation period longer than two years and a corresponding large sample size that increases the quality of the findings. Additionally, only studies regarding methacrylate-based composite resins were included, which enables the characterization of the specific material class. However, a limitation of this study is the low number of clinical trials that could be included. This might influence the outcome of the PICO question of this review.

## 5. Conclusions

With regard of the limited number of clinical trials and the short follow-up period of two years of four studies from a total of six included studies, a conclusion for practicing dentists is difficult. The use of flowable composites as a cavity liner does not appear to have any clinical effect on the clinical longevity of direct composite restorations. Further studies with a longer observation period are necessary to draw a clear conclusion. The disparate methodologies and measured parameters employed in the analyzed studies also presented a challenge in terms of comparing the results. However, the available data do not indicate any clinical disadvantage of the additional use of flowable composite materials as an intermediary layer. Furthermore, the evaluated studies may indicate the implementation of a flowable material as a liner, which constitutes a viable option within the scope of restorative therapy for daily clinical practice among dentists due to easier handling properties and the ability of realizing better marginal adaptation. This approach exhibits neither distinct advantages nor disadvantages in terms of the treatment and clinical longevity of composite restorations in Class-I and Class-II cavities. Therefore, the application of a flowable composite as a cavity liner is a possible option in everyday clinical practice of dental practitioners and has some positive but no negative aspects.

## Figures and Tables

**Table 1:** A summary of the inclusion and exclusion criteria of published clinical trials.

Inclusion Criteria	Exclusion Criteria
representing all search terms used	articles without apparent relevance
	case reports, case series, editorials, case reviews, reviews
clinical trials	laboratory studies
permanent teeth	primary teeth
class-I and class-II restorations	class-V restorations
composite resins	glass ionomer cements, provisional materials, and bulk-fill composites

## Data Availability

Not applicable.
